# Body Knowledge and Emotion Recognition in Preschool Children: A Comparative Study of Human Versus Robot Tutors

**DOI:** 10.3390/bs16010029

**Published:** 2025-12-23

**Authors:** Alice Araguas, Arnaud Blanchard, Sébastien Derégnaucourt, Adrien Chopin, Bahia Guellai

**Affiliations:** 1 Laboratoire Ethologie, Cognition, Développement, University Paris Nanterre, 92000 Nanterre, France; 2 Equipes Traitement de l’Information et Systèmes (ETIS) Laboratory, University Cergy, 95031 Cergy, France; 3 Smith-Kettlewell Eye Research Institute, San Francisco, CA 94115, USA; adrien.chopin@gmail.com; 4 Cognition, Langues, Langage, Ergonomie (CLLE) Laboratory, University Toulouse Jean Jaurès, 31058 Toulouse, France; 5 Institut Universitaire de France, 75231 Paris, France

**Keywords:** children, social robots, body, emotions, interaction

## Abstract

Social robots are increasingly integrated into early childhood education, yet limited research exists examining preschoolers’ learning from robotic versus human demonstrators across embodied tasks. This study investigated whether children (aged between 3 and 6) demonstrate comparable performance when learning body-centered tasks from a humanoid robot compared to a human demonstrator. Sixty-two typically developing children were randomly assigned to a robot or a human condition. Participants completed three tasks: body part comprehension and production, body movement imitation, and emotion recognition from body postures. Performance was measured using standardized protocols. No significant main effects of demonstrator type emerged across most tasks. However, age significantly predicted performance across all measures, with systematic improvements between 3 and 6. A significant age × demonstrator interaction was observed for sequential motor imitation, with stronger age effects for the human demonstrator condition. Preschool children demonstrate comparable performance when interacting with a humanoid robot versus a human in body-centered tasks, though motor imitation shows differential developmental trajectories. These findings suggest appropriately designed social robots may serve as supplementary pedagogical tools for embodied learning in early childhood education under specific conditions. The primacy of developmental effects highlights the importance of age-appropriate design in both traditional and technology-enhanced educational contexts.

## 1. Introduction

Contemporary preschool children are the first generation to develop in environments where artificial agents serve as increasingly sophisticated social partners. This raises fundamental questions about human–robot learning interactions and whether embodied artificial agents can complement traditional pedagogical approaches. Social robot deployment in educational contexts has increased substantially, with AI advancements showing measurable influences on children’s learning and development (Li et al., 2024). Understanding these dynamics is critical as educational institutions integrate AI technologies and because early childhood social learning establishes foundational competencies for complex social behaviors.

### 1.1. Social Learning and Selective Tutor Preference

Children acquire essential competencies through observational learning and selective imitation (Aksoy & Baran, 2010; Bandura, 1977). From infancy, children demonstrate sophisticated information-processing when observing different models (Bridgers et al., 2020; Guellai & Streri, 2022). This emphasizes active knowledge construction rather than passive mimicry in early development.

Social learning effectiveness depends on tutor characteristics. Children show « selective social learning », i.e., systematic preferences for information sources based on evaluative criteria. Before age five, children favor familiar individuals over knowledgeable strangers, using familiarity as a trust heuristic (P. E. Brosseau-Liard & Birch, 2010; Corriveau et al., 2009; Wood et al., 2017). This extends to voice familiarity, with enhanced learning from familiar voices (Montgomery et al., 2024). By six months, infants prefer their native language and native speakers, showing early sensitivity to social group membership that influences selective learning (Begus et al., 2016; Kinzler et al., 2007; Kinzler & Spelke, 2011). As children mature, they shift toward prioritizing expertise over familiarity. This reflects increasingly sophisticated reasoning about others’ knowledge states and the emergence of epistemic vigilance, the ability to critically assess information source reliability (Koenig & Harris, 2005; Pasquini et al., 2007).

Children also evaluate tutor competence, often prioritizing prior accuracy over intentions or social affiliation when deciding whom to trust (Einav & Robinson, 2010; Koenig & Harris, 2005; Zmyj et al., 2010). Despite this sensitivity, preschoolers exhibit strong default trust in adult informants, sometimes endorsing claims contradicting their own perceptions (Jaswal, 2010; Mills, 2013). Developing theory of mind and statistical reasoning support children’s ability to detect and respond to informational reliability (Bridgers et al., 2020; Gweon et al., 2014; Poulin-Dubois & Brosseau-Liard, 2016).

Additional factors shape learning preferences. Linguistic and cultural markers strongly influence tutor preference, with children favoring perceived in-group members (Somogyi et al., 2020). Children attend differently to peers, older children, and adults, with imitation varying by perceived authority (Perloff, 1982; Shutts et al., 2009). Physical attributes and behavioral cues further modulate engagement, potentially reflecting evolved heuristics for identifying trustworthy informants (P. Brosseau-Liard et al., 2014; Olson & Spelke, 2008). These selection processes align with Bandura’s (1977) social learning theory, emphasizing attention, retention, reproduction, and motivation. These findings highlight the complexity of children’s social learning strategies, particularly when considering non-human agents like social robots as potential learning partners.

### 1.2. Social Robots in Educational Contexts

Educational technologies have positioned social robots as potentially valuable pedagogical tools. Unlike conventional digital media, social robots provide embodied presence and operate with varying autonomy (van den Berghe et al., 2019). These systems exhibit human-typical behavioral norms (Bartneck & Forlizzi, 2004) while enabling natural engagement through multimodal communication including touch, gesture, and vocal interaction (Robinson et al., 2019). The humanoid NAO robot appears in 48% of child-robot interaction studies (Belpaeme et al., 2018), with applications spanning mathematical instruction and creative storytelling (Kim et al., 2013; Maure & Bruno, 2023).

Empirical findings regarding robot effectiveness present mixed results, partly reflecting embodiment’s complex role in children’s learning. Physical presence and embodied interactions appear crucial for optimal learning, as children rely on multimodal cues including gesture, facial expressions, and spatial positioning to guide attention and comprehension (Alibali & Nathan, 2012; Goldin-Meadow, 2003). Comparative studies show higher child engagement with human versus robot instructors in preschool settings, potentially due to humans’ richer embodied communicative repertoire (Ciornei et al., 2023). Bodily co-presence facilitates joint attention, gesture comprehension, and social synchronization supporting knowledge acquisition (Kuhl et al., 2003; Meltzoff et al., 2009; Roseberry et al., 2014). Conversely, research demonstrates robots’ potential to provide engaging, non-judgmental contexts promoting skills like reading engagement, suggesting carefully designed embodied robot behaviors can partially compensate for limitations (Akinola et al., 2023). However, children’s reliance on physical cues and spatial cognition continues evolving throughout early childhood, influencing information processing from different learning partners (Mix & Cheng, 2012; Newcombe & Frick, 2010). This underscores the necessity of identifying specific conditions under which robots can effectively complement human instruction within embodied learning frameworks.

### 1.3. Embodied Cognition and Body-Centered Learning

Social robots offer unique opportunities for investigating embodied learning within the theoretical framework of embodied cognition, which emerged prominently in the early 90s through seminal works by Varela et al. (1991) and later developments by Clark (1997) and Lakoff and Johnson (1999). This perspective posits that cognitive processes are fundamentally grounded in bodily experience. Physical actions, sensorimotor processes, and environmental interactions are not auxiliary to cognition but constitute its foundation (Barsalou, 2008; Glenberg, 2010; Wilson, 2002). The embodied cognition framework suggests abstract concepts are understood through metaphorical extensions of bodily experiences, with motor systems playing crucial roles in language comprehension and conceptual processing (Fischer & Zwaan, 2008; Gallese & Lakoff, 2005). Recent advances in embodied AI highlight the integration of perceptual, cognitive, and behavioral capabilities (Li et al., 2024), with growing recognition of embodied cognition’s unifying potential in AI development and human–robot interaction (Clark et al., 2024; Pfeifer & Bongard, 2006).

Body knowledge represents a particularly compelling domain for robot-mediated embodied learning research. It directly engages embodied cognition principles through self-referential, physically grounded learning experiences. The human body serves as the primary reference point for spatial cognition, motor planning, and social understanding, making body knowledge acquisition an ideal testing ground for embodied learning theories (de Vignemont, 2010; Gallagher, 2005). Recent evidence demonstrates children can acquire knowledge from social robots under specific conditions, particularly when robots exhibit interactive, contingent, and socially appropriate behaviors mirroring natural pedagogical interactions (Belpaeme et al., 2018; Tsutsui et al., 2024). Bodily representations encompass three distinct but interconnected categories: dynamic body schema for motor control and action planning, visuo-spatial body mapping for topographical relationships, and lexicosemantic representation including body part nomenclature and conceptual understanding (Longo et al., 2010; Raimo et al., 2021; Schwoebel et al., 2004).

Developmental research using the established Bergès and Lézine somatognosia assessment reveals systematic improvements in body part label comprehension and production among children aged 3–6 years, with comprehension consistently exceeding production—a pattern consistent with broader psycholinguistic principles (Bergès & Lézine, 1963; Fenson et al., 1994; Russo et al., 2018). Children’s body part knowledge follows predictable trajectories: facial features and prominent body parts (head, arms, legs) are identified before distal elements and joints, with recognition patterns dependent on cortical sensory representation density, frequency of adult naming, functional salience, and visual accessibility (Camões-Costa et al., 2011; Naito & Ehrsson, 2006; Penfield & Rasmussen, 1950). However, robot-based body knowledge research has predominantly focused on therapeutic applications for children with Autism Spectrum Disorder (ASD) (Diehl et al., 2012), with limited systematic investigation of typically developing children’s comparative learning performance across robot and human demonstrators.

### 1.4. Bodily Expression Recognition in Learning Contexts

Understanding bodily expressions constitutes a crucial aspect of embodied learning, as these expressions serve as fundamental channels for emotional and social communication supporting pedagogical interactions. Facial and bodily expressions function as complementary information channels, with bodily cues often providing more robust emotional signals than facial expressions alone, particularly for high-arousal emotions like anger and fear (Aviezer et al., 2012; Meeren et al., 2005). Children aged 3–6 years can recognize primary emotions through both facial and bodily channels, with systematic improvements in accuracy and speed throughout this period (Boone & Cunningham, 1998; Widen & Russell, 2003). However, bodily emotion recognition typically develops later than facial recognition, with children showing adult-level performance for bodily expressions around age 6–7 years compared to age 4–5 for facial expressions (Lagerlöf & Djerf, 2009; Mondloch, 2012).

This developmental trajectory assumes particular educational significance during early childhood, when nonverbal communication often compensates for limited verbal abilities and children rely heavily on multimodal emotional cues to understand social learning contexts (Izard et al., 2001; Ruba & Pollak, 2020). The ability to interpret instructor emotions through bodily expressions directly impacts children’s attention, motivation, and learning outcomes (Mega et al., 2014; Pekrun et al., 2002). Recent findings indicate 5-year-old children demonstrate sophisticated social cognition when interacting with social robots, showing reputation concerns and attributing enhanced properties to interactive robots, suggesting children’s emotional interpretation of robot behavior may influence learning receptivity (Kahn et al., 2012; Okumura et al., 2013).

Research using the NAO robot demonstrates that adults and adolescents successfully recognize basic emotions from static robotic body postures, even without facial expressions, indicating simplified embodied emotional displays retain communicative effectiveness (Beck et al., 2010, 2013). Subsequent investigations confirm that NAO robots can convey emotional states through dynamic body movements and gestures, with recognition accuracy varying by emotion type, happiness and sadness being more readily identified than anger or fear (Cohen et al., 2012; Stock-Homburg et al., 2018). Contemporary applications include NAO robots facilitating mathematical concept acquisition through embodied interactions incorporating emotional engagement and motivational feedback (Alves-Oliveira et al., 2019; Fong et al., 2023). However, these investigations predominantly involved older children, adolescents, and adults, creating a research gap regarding preschool children’s interpretation and learning responses to emotional expressions from robotic versus human agents. Given that younger children show different developmental patterns in emotion recognition and may rely more heavily on multimodal emotional cues, their responses to robot emotional expressions likely differ qualitatively from older populations (Herba & Phillips, 2004; Widen, 2013).

### 1.5. Study Rationale and Objectives

The present study addresses critical gaps in understanding how typically developing preschool children (aged between 3 and 6) engage with embodied artificial agents across body-centered learning tasks. We conducted a controlled comparison between the NAO robot and a human demonstrator across two key domains: body knowledge (comprehension, production, and imitation) and emotion recognition from body postures.

Our investigation, guided by embodied cognition theory, addresses two primary research questions:

(1) Do preschool children demonstrate comparable learning and recognition performance when interacting with social robots compared to human demonstrators in body-centered tasks?
(2) How do developmental factors modulate children’s responsiveness to different agent types?

We hypothesized superior performance in the human compared to the robot condition, reflecting humans’ richer embodied communicative repertoire, and the greater morphological similarity in body schema between two humans compared to a human and an anthropomorphic robot. However, we expected robots to still function as effective learning partners, albeit with reduced effectiveness compared to human instruction. Additionally, we predicted age-related performance improvements across both conditions, consistent with established developmental trajectories in body knowledge and emotion recognition. Through systematic experimental comparison of robot and human instructors across a comprehensive battery of bodily knowledge and emotion recognition tasks, this study provides empirical evidence to guide principled design and implementation of robot-assisted learning technologies in early childhood educational contexts. By examining preschool children’s learning responses to embodied instruction from both human and robotic agents, our investigation tests theoretical predictions derived from embodied cognition frameworks and advances our understanding of conditions under which artificial agents may effectively support children’s developmental learning processes.

## 2. Methods

### 2.1. Participants

Seventy-three children, aged from 3 years 3 months to 6 years 1 month, were evaluated. All children were French native speakers from 4 different schools. We obtained parental consent for all children and collected verbal assent from the participants before starting.

Of 73 children initially recruited, 9 asked to stop before completion as they did not feel comfortable in this unfamiliar situation, and 2 experienced technical camera issues. In total, 62 child-demonstrator interactions were completed in a between-subject design (school A: 7 children, school B: 10 children, school C: 17 children, school D: 28 children).

Children were separated into three age groups:

- Group 1 (G1): 3 years 1 month to 4 years 1 month (mean ± SE = 3.73 ± 0.07).
- Group 2 (G2): 4 years 2 months to 5 years 1 month (mean ± SE = 4.71 ± 0.06).
- Group 3 (G3): 5 years 2 months to 6 years 1 month (mean ± SE = 5.65 ± 0.059).

We balanced experimental groups for age and gender. Children were randomly assigned to conditions. Our sample comprised 62 children (30 robot group, 32 human group). G1: 14 children (8 girls: 4 robot, 4 human; 6 boys: 2 robot, 4 human). G2: 26 children (13 girls: 7 robot, 6 human; 13 boys: 6 robot, 7 human). G3: 22 children (14 girls: 6 robot, 8 human; 8 boys: 5 robot, 3 human). All children were typically developing.

The protocol followed ethical standards of the Declaration of Helsinki and was approved by the Ethics Committee of the Department of Psychology (CER-PN n°2022-09-01).

### 2.2. Experimental Procedure

#### 2.2.1. General Setup

**Figure 1:**
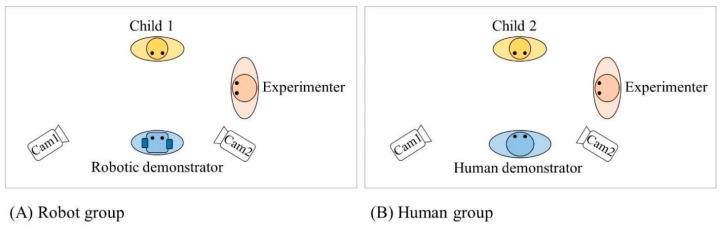
Top view of the experimental setup for the two groups: (**A**) with the robotic demonstrator and (**B**) with the human demonstrator (inspired by Kennedy et al., 2015). The child and the demonstrator sat in front of each other and the experimenter was on the demonstrator’s right side.

Experimental sessions were conducted in a quiet school room with the experimenter and demonstrator (Figure 1). In each school, one adult female experimenter was recruited and trained. No child had prior NAO robot exposure.

The experimenter asked the child to sit facing the demonstrator. The demonstrator was a female adult for half the children and the NAO robot for the other half. Due to COVID-19, the experimenter and human demonstrator wore face masks throughout. Each session lasted approximately 20 min. Two digital video cameras (1920 × 1080 resolution, 30 fps) on tripods from either side of the demonstrator recorded child behaviors.

Two conditions were designed:

(1) Human group: Exposure to an adult demonstrator

The human demonstrator was always the same adult female who remained as neutral as possible. She gave instructions but did not encourage or provide feedback.

(2) Robot group: Exposure to the NAO robot demonstrator.

**Figure 2:**
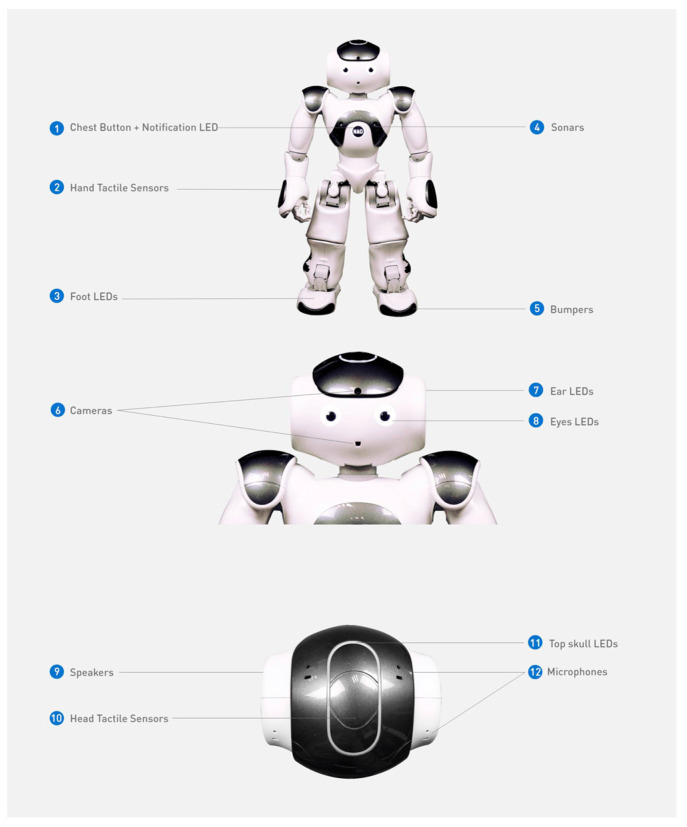
Example of the design of the NAO robot.

The NAO robot version 6 (Aldebaran Robotics) is 58 cm high and bipedal with 25 degrees of freedom allowing diverse movements (see Figure 2). It can manipulate small objects with three-finger hands. NAO has two video cameras on forehead and mouth. It has vocal capacities for recognition and synthesis with a stereo broadcast system of two loudspeakers on ears and 4 omnidirectional microphones (2 on head top, 2 on head back). It has two ultrasonic sensors (sonars) estimating distance to obstacles. It has contact and tactile sensors: tactile head and hands, chest button, and feet bumpers. NAO can be programmed for autonomous tasks. However, for our experiment, the robot was fully teleoperated for contingent action: the experimenter controlled the robot via touchscreen tablet (20.32 × 13.48 × 0.61 cm). Thanks to a robotic engineer who programmed the robot, the setup was connected from the tablet to the robot’s wifi access point. The robot hosted a web server accessible through any browser on the tablet. An HTML page provided the interface and executed the experiment’s sequence using javascript (vue.js for graphical interface, LibQi for robot control).

We attributed feminine gender to the NAO robot with a feminine name (“Naomie”) matching the female human demonstrator. To ensure both demonstrators pronounced identical sentences, vocal recordings of the female adult were played back by the robot. Sentences were recorded alone in a silent room addressing a child.

#### 2.2.2. Experimental Tasks and Scores

Before starting experimental tasks, a warm-up phase allowed children to see how the demonstrator spoke and moved. The demonstrator said her name and asked the child to introduce themselves. She then sang a popular rhyme with hand gestures and encouraged the child to follow.

Each child was tested in different situations: (a) comprehension of body part labels on their own body and on the demonstrator’s body, (b) production of body part labels and an imitation task, and (c) identification of emotional key postures.

(a) Comprehension of Body Part Labels

We evaluated the comprehension of eleven body part labels: face, eye, nose, mouth, ear, shoulder, elbow, hand, belly, knee, foot. Each body part was randomly chosen. First, the demonstrator asked “Show me your [body part]” and repeated for eleven possibilities. The child obtained 1 point for each body part correctly shown (maximum 11 points for identification on their own body). Then, she asked “Show me my [body part]” and repeated for eleven body parts. The child obtained 1 point for each body part correctly indicated on the demonstrator’s body (maximum 11 points). Coders verified whether the child touched or pointed to the correct body part in response to each verbal prompt. Percentage score was calculated as: (number of correct responses/11) × 100.

(b) Imitation of Body Part Sequences

We evaluated body part label production: the demonstrator randomly showed one of her body parts (e.g., her eye) and asked “What is the name of this body part?” and a second one (e.g., her belly) and said “And this one?”. Each correct verbal label was scored as 1 point (maximum 9 points). Percentage was calculated as: (correct responses/9) × 100.

Since it was complicated for the NAO robot to point to her nose and ear, we kept 9 body parts for this sub-task (face, eye, mouth, shoulder, elbow, hand, belly, knee, foot).

Then, the demonstrator placed her hands on her knees and asked the child to repeat the body part sequence. Each body part correctly reproduced in the correct sequential position received 1 point.

The demonstrator performed two sequences with two body parts (“sequence#1”, “sequence#2”) and a third sequence with three body parts (“sequence#3”). Since most children had difficulty understanding the instructions, the first two-body-part sequence (sequence#1) was used to re-explain instructions. Sequence #1 was excluded from analysis as it served as a practice trial. The combined score from sequences #2 (2 body parts) and #3 (3 body parts) yielded a maximum of 5 possible points. Percentage was calculated as: (correct responses/5) × 100.

(c) Emotions Task

Four images of a child expressing an emotion (joy, sadness, anger, fear) were placed before the child. The demonstrator asked “Do you know what an emotion is? Show me the image in which the child feels [emotion]” (see Supplementary Material). Each correct identification = 1 point (maximum 4 points). Percentage: (correct responses/4) × 100.

Then, the demonstrator and child stood up. The demonstrator asked “Show me how you express [emotion] with your whole body”. She waited for the child to express the emotion. The instruction was repeated with four emotions. Scoring criteria: full-body expression (movement involving trunk, limbs, and posture) = 1 point; facial expression only (no body movement) = 0.5 points; no movement or verbal description only = 0 points (maximum 4 points). Percentage: (total points/4) × 100.

Since the NAO robot lacks facial expressions, we investigated emotion recognition when emotions were expressed with the body. We selected four key postures exhibited by the NAO robot most successfully identified by adults and children in previous studies (Beck et al., 2010, 2013): joy, fear, anger, and sadness. The demonstrator struck each of these four key postures and between each pose, returned to a neutral pose (see Supplementary Material).

For this task, the demonstrator said « Let me show you what I am doing when I am feeling an emotion ». The demonstrator reproduced one of four emotional postures and the experimenter asked “Which emotion do you think it is?”. After the child replied, the demonstrator returned to the neutral pose. She repeated this sequence for three other body postures. Each correct identification = 1 point (maximum 4 points). Percentage: (correct responses/4) × 100. At session end, the demonstrator thanked the child for participation and said goodbye.

#### 2.2.3. Coding Procedure and Reliability

All experimental sessions were video recorded using two digital video cameras (1920 × 1080 resolution, 30 fps) to capture the child’s face and body movements (Figure 1). Video recordings were subsequently coded offline by a trained researcher (AA) who viewed the footage and scored children’s responses according to predetermined criteria for each task. To establish inter-rater reliability, 20% of videos (12 randomly selected sessions) were independently coded by a second trained researcher blind to study hypotheses. Inter-rater agreement was high (Cohen’s kappa = 0.92), indicating excellent coding scheme reliability. Discrepancies were resolved through discussion and consensus.

Informed written consents were obtained from the human demonstrator and children’s parents for video recordings and to publish information/images in an online open-access publication.

## 3. Results

We tested whether children of different ages would obtain better performances when interacting with a human rather than a robot. To answer that question, we conducted Generalized Linear Models (GLM) and Generalized Linear Mixed Effects Models (GLME) with the following factors as fixed-effect predictors: the effects of demonstrator type (robot vs. human), the expected effect of the child’s age (continuous variable in years), with older children obtaining better performance across all tasks, the interaction between age and demonstrator type and a possible effect of child’s gender.

Our model selection procedure was as follow: we began with GLME models including school and experimenter as random effects to account for potential clustering within schools and variation across experimenters. None of the dependent variable was normally distributed (all Shapiro tests *p* < 0.05), so we used Gamma distributions, adding a small number to avoid 0 values. When the distribution was away from zero, we transformed the variable (e.g., the number of correct responses was transformed into the number of errors). We systematically evaluated different link functions (reciprocal, identity, log) and selected the link with the smallest AIC values (Akaike Information Criterion). When including of random effects did not improve model fit (indicated by higher AIC values), we simplified the GLME to a GLM.

All statistical analyses were conducted in MATLAB (R2023b). These functions use the residual method to define the degrees of freedom (df = n – p with n = 62 observations and *p* = 5 fixed-effects factors including intercept). Statistical significance was set at α = 0.05 for all tests.

Power analysis

We conducted a post hoc power analysis using G*power 3.1.9.7 considering the t-tests family for a linear multiple regression: fixed model, single regression coefficient, at alpha level 5%. With our sample of 62 subjects and 5 predictors, we could detect one-tail medium effect sizes (f^2^ = 0.15) with a power of 91% but we had only a power of 29% for detecting small-sized effects (f^2^ = 0.02).

Regarding GLMEs, we assumed that GLMEs increase our power in comparison to GLMs by accounting for nuisance effects in the intercepts. Our GLMEs have then at least the power that we calculated for the GLMs. Therefore, we checked that all our null hypothesis results would stand if we had only conducted a GLM instead and it was the case.

### 3.1. Comprehension of Body Part Labels

**Figure 3:**
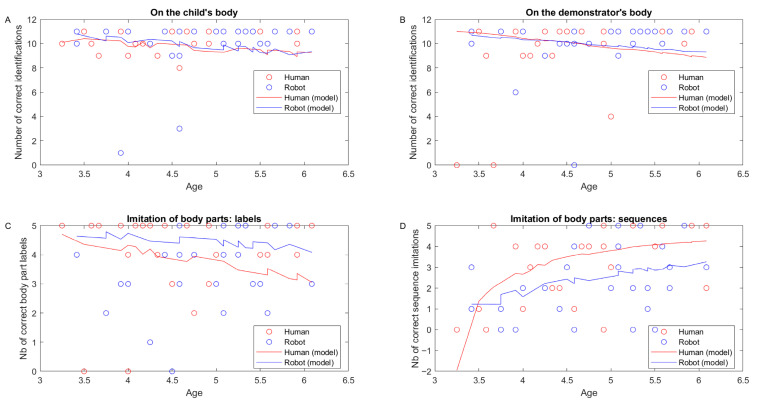
Correct identifications in the body part task depending on child’s age for the robot group (blue circles) and the human group (red circles) on (**A**) the child’s body or (**B**) the demonstrator’s body. (**C**) Correct body part labels when naming body parts as a function of the child’s age for the robot group (blue circles) and the human group (red circles). (**D**) Correct body part sequences imitated as a function of the child’s age for the robot group (blue circles) and the human group (red circles). Each data point represents one child’s performance. Model estimates from GLM/GLME are shown for each group (robot: blue line, human: red line). Panel D shows the significant age × demonstrator interaction effect.

We used a GLM with log link on the number of errors in body identifications on the child’s body and re-transform the data in the figure and analysis below to express the number of correct body identifications on the child’s body. This number did not vary with our predictors (age: t(57) = −1.22, *p* = 0.22; type of demonstrator: t(57) = 1.79, *p* = 0.07; gender: t(57) = 0.01, *p* = 0.98; interaction age × demonstrator: t(57) = −1.75, *p* = 0.08; Figure 3A).

We used a GLM with identity link on the number of errors in body identifications on the demonstrator’s body and re-transform the data in the figure and analysis below to express the number of correct body identifications on the demonstrator’s body. This number increased with age (t(57) = −2.86, *p* = 0.005) but did not vary significantly with other predictors (type of demonstrator: t(57) = −0.56, *p* = 0.57; gender: t(57) = −1.00, *p* = 0.31; interaction age × demonstrator: t(57) = 0.55, *p* = 0.58; Figure 3B).

### 3.2. Imitation of Body Part Sequences

For the imitation of body part sequences, we only used sequences of two or three body parts as it was necessary to use the first trial (one body part) to re-explain instructions. We used a GLM with identity link on the number of errors in body parts labelled (production of body part labels) and re-transform the data in the figure and analysis below to express the number of body parts correctly labelled. This number significantly increased with age (t(57) = −3.13, *p* = 0.002) but did not vary significantly with type of demonstrator (t(57) = 0.35, *p* = 0.72), gender (t(57) = 1.69, *p* = 0.09), or interaction age × demonstrator (t(57) = −0.04, *p* = 0.96; Figure 3C).

We used a GLME with reciprocal link on the number of errors in body parts imitated in sequences and re-transformed the data in the figure and analysis below to express the number of body parts correctly imitated. This number significantly increased with age (t(57) = 3.78, *p* < 0.001) but did not vary significantly with type of demonstrator (t(57) = 1.70, *p* = 0.09) or gender (t(57) = −0.41, *p* = 0.68). However, the effect of age was stronger for the human demonstrator than for the robot demonstrator (significant demonstrator x age interaction: t(57) = −2.06, *p* = 0.043; Figure 3D).

### 3.3. Emotions Task

**Figure 4:**
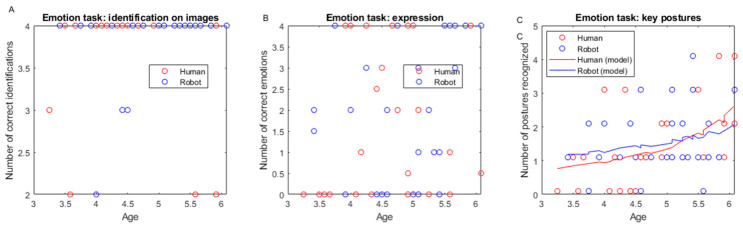
(**A**) Number of emotions correctly identified on images depending on child’s age for the robot group (blue circles) and the human group (red circles). (**B**) Number of emotions correctly expressed depending on child’s age for the robot group (blue circles) and the human group (red circles). (**C**) Number of key postures correctly recognized depending on child’s age for the robot group (blue circles) and the human group (red circles). Model estimates from GLM/GLME are shown for each group (robot: blue line, human: red line). For panels A and B, there are no model estimates shown because the best model was not significantly different from the constant model. Each data point represents one child’s performance.

We used a GLM with log link on the number of errors in emotions identified on images and re-transform the data in the figure and analysis below to express the number of correct emotions identified on images. This number did not vary with our predictors (age: t(57) = −0.05, *p* = 0.95; type of demonstrator: t(57) = 1.38, *p* = 0.17; gender: t(57) = −0.03, *p* = 0.97; interaction age × demonstrator: t(57) = −1.57, *p* = 0.12), and the model was not significantly different from a constant model (*F* = 1.04, *p* = 0.393; see Figure 4A).

We used a GLM with reciprocal link on the number of errors in emotions expressed by the child and re-transform the data in the figure and analysis below to express the number of correct emotions expressed by the child. This number did not vary significantly with our predictors (age: t(57) = 1.63, *p* = 0.10; type of demonstrator: t(57) = 0.61, *p* = 0.54; gender: t(57) = 1.22, *p* = 0.22; interaction age × demonstrator: t(57) = −0.64, *p* = 0.51, see Figure 4B).

We used a GLM with reciprocal link on the number of errors in emotional key postures recognized and re-transform the data in the figure and analysis below to express the number of correct emotional key postures recognized. We observed an effect of children’s age for identification of emotional key postures: the number of emotional key postures recognized by younger children was lower compared to older ones (t(57) = −2.76, *p* = 0.007) but did not vary significantly with other predictors (type of demonstrator: t(57) = −1.07, *p* = 0.28; gender: t(57) = 0.36, *p* = 0.17; interaction age × demonstrator: t(57) = 1.02, *p* = 0.31; see Figure 4C).

## 4. Discussion

This study provides the first systematic evidence that typically developing children aged 3–6 years demonstrate comparable performance when interacting with NAO humanoid robots versus human demonstrators across fundamental developmental assessment tasks. These findings have significant methodological and practical implications for developmental research and educational technology design.

### 4.1. Core Empirical Findings

Children showed a critical dissociation between self-directed and other-directed body part identification in body schema processing tasks. They demonstrated ceiling effects for self-identification (consistent with mastery by ages 2–3; Camões-Costa et al., 2011), while identification on external demonstrators improved significantly with age, regardless of demonstrator type. This pattern reflects the complex cognitive demands of allocentric spatial processing and perspective-taking (Newcombe & Frick, 2010; Pisella et al., 2019). Crucially, developmental trajectories were comparable for human and robot body schemas, suggesting children process robotic embodiment through the same allocentric spatial mechanisms used for human bodies.

The significant age × demonstrator interaction in sequential motor imitation provides critical evidence against interpreting our findings as simple equivalence. Younger children (3–4 years) performed comparably across conditions, but older children (5–6 years) showed steeper improvements with human demonstrators. This pattern indicates that (1) underlying cognitive processes differ between demonstrator types despite comparable performance levels, (2) morphological familiarity becomes increasingly advantageous as imitative capacities mature, and (3) children may achieve similar outcomes through different cognitive strategies depending on demonstrator type. This task-specific effect was absent in body part identification and emotion recognition, suggesting demonstrator morphology’s influence varies by cognitive demand.

Children achieved ceiling effects for facial emotion recognition by age 3 in emotional processing tasks, consistent with established timelines (Ekman & Friesen, 1976; Widen & Russell, 2003). However, body posture emotion recognition continued developing throughout the tested age range, independent of demonstrator type. This dissociation highlights the extended developmental timeline for integrating complex bodily emotional cues (Mondloch, 2012). Substantial individual variability in emotional expression abilities remained independent of age and demonstrator type, suggesting personality factors (extraversion, emotional expressiveness) influence performance more than interaction partner characteristics (Riggio & Riggio, 2002).

### 4.2. Theoretical Implications

Our findings demonstrate what we term «cognitive flexibility», children’s capacity to adaptively extract relevant information from fundamentally different embodied forms (Wykowska, 2020). This differs from «robustness»: rather than maintaining identical processing mechanisms despite variations, children appear to flexibly deploy appropriate cognitive strategies depending on agent characteristics. The motor imitation interaction effect supports this interpretation.

These results challenge morphological similarity theories proposing children learn more effectively from agents sharing their physical characteristics (Jones, 2009; Meltzoff & Moore, 1977) and concerns about uncanny valley effects impeding child-robot interaction (Mori et al., 2012). Instead, they support domain-general theories of social learning emphasizing representational flexibility over perceptual similarity (Csibra & Gergely, 2009; Tomasello et al., 2005).

For embodied cognition theories, our findings suggest spatial, motor, and social cognitive processes operate at a more abstract level than traditional accounts propose (Barsalou, 2008; Wilson, 2002). Children’s spatial cognitive systems appear to prioritize functional and relational properties over specific morphological features when processing body schemas.

### 4.3. Methodological Implications

Our findings have direct implications for developmental assessment methodology. The comparable performance across most tasks suggests appropriately designed robots could serve as standardized assessment tools, offering several methodological advantages. Robots eliminate assessor variability in presentation, timing, and emotional expression, common sources of measurement error in developmental research. Robotic demonstrators across timepoints could provide unprecedented precision in tracking developmental changes. Moreover, replicable demonstrations may improve inter-rater reliability and cross-site comparability, and consistent robot contexts may reveal individual differences more clearly than variable human interactions.

However, the motor imitation interaction effect indicates demonstrator type may differentially affect performance depending on developmental stage and task characteristics. Therefore, researchers must carefully consider which assessment domains are appropriate for robot administration and validate findings against human-administered protocols.

### 4.4. Practical and Educational Implications

Our findings do not suggest robots should replace human educators. Preschool years are foundational for socioemotional development, and warm, responsive human relationships remain irreplaceable for children’s holistic development. Rather, under specific, carefully controlled conditions, appropriately designed robots may serve as supplementary tools complementing human instruction.

Potential applications include inclusive education contexts where robots may provide consistent, predictable learning partners for children with special educational needs (Ghiglino et al., 2025; Scassellati et al., 2012). In multilingual contexts, robots could offer consistent language models where linguistic diversity creates instructional challenges. For standardized assessment, robots may reduce assessor bias and increase measurement precision in developmental evaluation.

However, thoughtful adaptation is essential. Cultural attitudes in socio-cognitive development vary substantially with age (Somogyi et al., 2020), and our sample represents only one cultural context. Children with diverse developmental profiles may respond differently than our typically developing sample. Therefore, inclusive design must involve stakeholders from target communities to ensure cultural appropriateness and pedagogical effectiveness.

### 4.5. Limitations and Future Directions

We operationalize «comparable performance» as the absence of statistically significant main effects of demonstrator type on task accuracy. However, this does not imply identical cognitive processes, as evidenced by the significant age × demonstrator interaction in sequential motor imitation.

We acknowledge some limitations in statistical power. Post hoc analysis (see Supplementary Analysis) indicates our sample size could detect medium effect sizes (f^2^ > 0.15) but not small effects (f^2^ > 0.02). Small differences between conditions may exist that our models failed to detect, necessitating replication with larger samples.

In addition, our findings emerged from brief, structured assessment contexts with typically developing children. Several moderating variables warrant future investigation: technological familiarity, child and robot gender, functional diversity, cultural context, and interaction duration. We specifically note that COVID-19 protocols required human demonstrators to wear masks, potentially reducing their natural communicative advantage and minimizing condition differences. In addition, our cross-sectional design cannot capture individual developmental trajectories, which require longitudinal investigation in future studies. Another interesting perspective could be to propose stakeholders workshops introducing the NAO in order to provide transparency about the research process, addressing potential concerns about robot use in educational contexts, and gathering valuable input from educators and parents about appropriate and acceptable applications of educational robotics.

## 5. Conclusions

From a methodological perspective, our findings suggest appropriately designed robots could serve as valuable standardized assessment tools, offering improved control over assessor variability while tracking developmental changes with unprecedented precision. However, careful validation against human-administered protocols remains essential, particularly for tasks involving complex motor imitation.

From a practical perspective, our results inform evidence-based approaches to integrating social robots as supplementary educational tools. Rather than replacing human educators, robots may complement instruction in specific contexts, particularly standardized assessment, inclusive education, and multilingual settings, when designed with careful attention to task demands and developmental appropriateness.

As artificial agents become increasingly prevalent in educational settings, understanding the conditions under which they can effectively support children’s natural learning processes becomes crucial. This empirical foundation enables educators, researchers, and policymakers to make informed decisions about educational technology implementation based on scientific evidence rather than technological enthusiasm or skepticism, ultimately supporting the thoughtful integration of artificial agents as supplementary tools in specific developmental contexts.

## Data Availability

We declare that data collected will be available upon request to B.G.

## Supplementary Materials

The following supporting information can be downloaded at: https://www.mdpi.com/article/10.3390/bs16010029/s1, Figure S1: The 9 body parts pointed by the demonstrator in the imitation task: (A) human and (B) robotic; Figure S2: Images used in the task of emotions’ identification (source accessed 1 January 2024: https://fr.freepik.com/); Figure S3: The neutral and emotional postures exhibited by the demonstrator: (A) human and (B) robot; Supplementary Analysis: Power Analysis.
