# Supplementary material for: Body Knowledge and Emotion Recognition in Preschool Children: A Comparative Study of Human Versus Robot Tutors
Source: Behav Sci (Basel). 2025 Dec 23;16(1):29. doi: 10.3390/bs16010029 (PMC12837169; doi:10.3390/bs16010029)

Supplementary Information

Supplementary Figure S1. The 9 body parts pointed by the demonstrator in the imitation task: (A) human and (B) robotic.

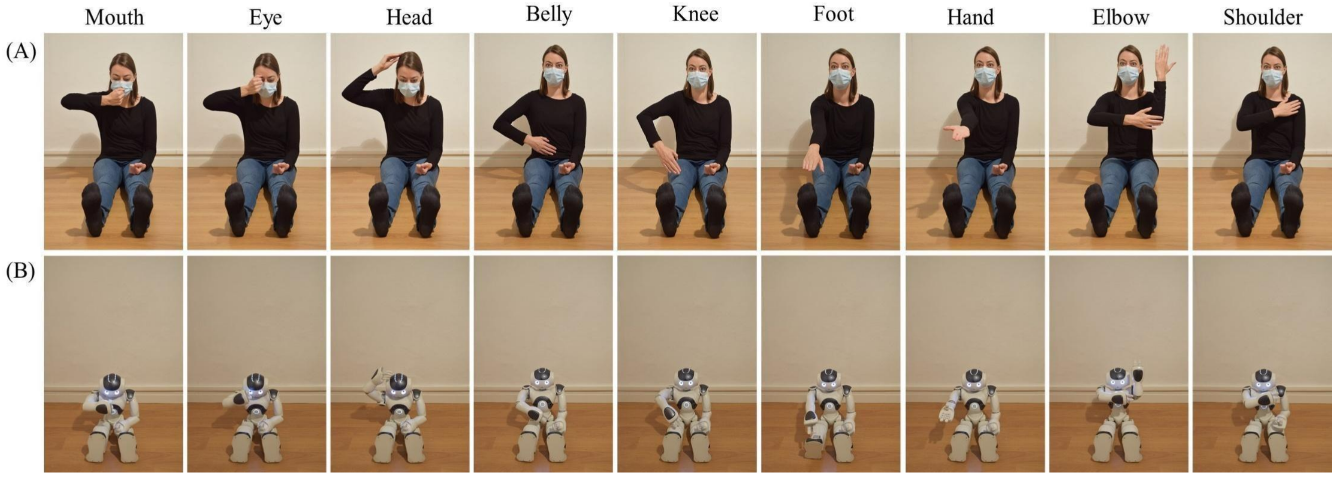

Supplementary Figure S2. Images used in the task of emotions' identification (source: https://fr.freepik.com/)

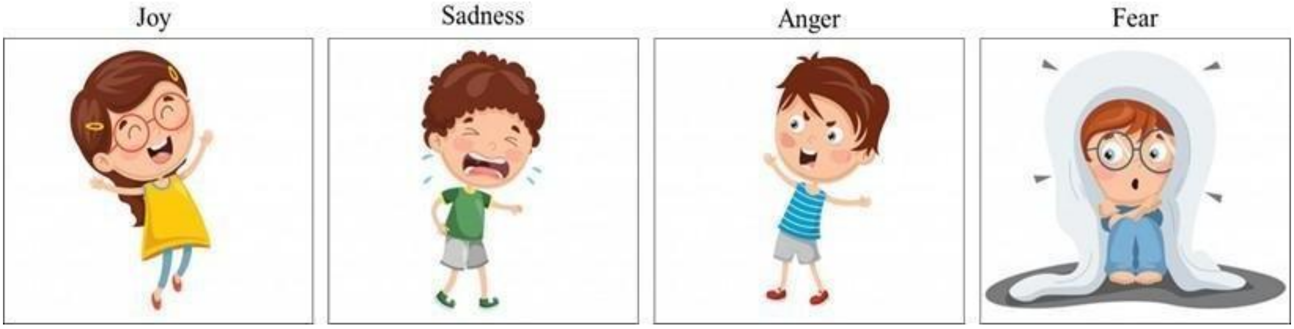

Supplementary Figure S3. The neutral and emotional postures exhibited by the demonstrator: (A) human and (B) robot.

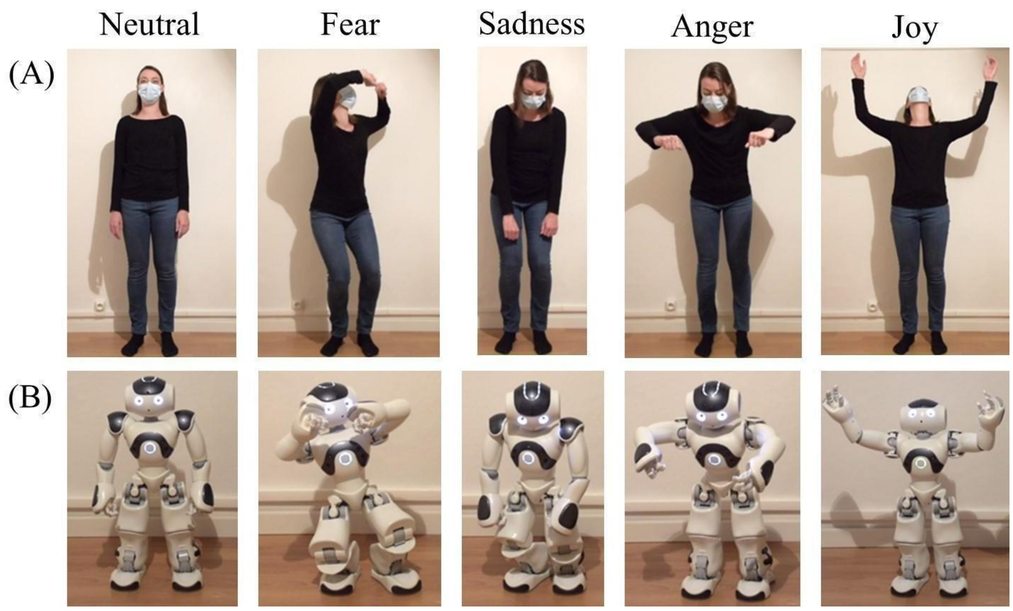

Supplement: Supplementary file 1 [file behavsci-16-00029-s001.zip › behavsci-3842849-supplementary.pdf]
